# Low-grade albuminuria in adult and elderly individuals with diabetes mellitus and arterial hypertension accompanied by Primary Health Care

**DOI:** 10.1038/s41598-021-96652-6

**Published:** 2021-09-02

**Authors:** Matheus Augusto Soares de Resende, Emily de Souza Ferreira, Heloísa Helena Dias, Daniel Souza Santos, Clara Regina Santos Batistelli, Luiza Delazari Borges, Luma de Oliveira Comini, Tiago Ricardo Moreira, Glauce Dias da Costa, Eunice Ferreira da Silva, Rodrigo Gomes da Silva, Rosângela Minardi Mitre Cotta

**Affiliations:** 1 Department of Nursing and Medicine, Federal University of Viçosa, Viçosa, Minas Gerais Brazil; 2 Department of Nutrition and Health, Federal University of Viçosa, Viçosa, Minas Gerais Brazil

**Keywords:** Biomarkers, Health care

## Abstract

Diabetes mellitus (DM) and arterial hypertension (AH) are the two main clinical conditions related to Chronic Kidney Disease (CKD); disease also identify by the levels of low-grade albuminuria (LGA). Few studies have simultaneously investigated the associations of glycated hemoglobin (HbA1c) and fasting plasma glucose (FPG) with LGA. Our study aimed to investigate and compare the association of HbA1c and FPG with the probability of LGA in adult and elderly individuals with DM and AH, within the scope of Primary Health Care (PHC). Cross-sectional study involving 737 Brazilians individuals previously diagnosed with hypertension and/or diabetes. Tests for HbA1c, FPG and LGA were performed. LGA was defined as the highest quartile of albumin urinary (≥ 13 mg/g) among individuals with urinary LGA < 30 mg / g. A significant increase in the prevalence of LGA was found with increasing levels of HbA1c (*p* < 0.001). There was a significant association of HbA1c with LGA (*p* < 0.001) and increased probability of LGA for participants with HbA1c ≥ 6.5% compared to those with Hba1c < 5.7% (OR [95% CI]: 2.43 [1.32–4.46], *p* < 0.05), after adjusting for confounding factors, except when adjusted for FPG (*p* = 0.379 and *p* = 0.359, respectively). HbA1c and FPG were significantly associated in a collinear manner with an increased probability of LGA in adult and elderly individuals with DM and AH.

## Introduction

The low-grade albuminuria (LGA) is considered an important predictor of Chronic Kidney Disease (CKD) and it is categorized as moderately increased albuminuria when its values are between 30 and 300 mg/g and severely increased above 300 mg/g^1^. Recent evidence shows that LGA, which is when urinary Urine Albumin-to-Creatinine Ratio (UACR) values are below the 30 mg/g threshold, is an early marker of cardiovascular mortality^2^, although albuminuria values at this level are considered normal^1^. In addition, urinary LGA was independently associated with glycated hemoglobin (HbA1c)^3^, whose values indicate the mean level of blood glucose in the last 3 months with less variability, despite fasting plasma glucose (FPG) being the test most commonly performed in screening for the diagnosis of diabetes mellitus (DM)^4^.

The worldwide prevalence of CKD was 9.1% in 2017 and, in Brazil, arterial hypertension (AH) and DM were the two main clinical conditions related to the evolution to dialysis, accounting for, respectively, 34% and 31% of primary causes of end-stage kidney disease (ESKD)^5,6^.

There are studies have evaluated the relationship between HbA1c and FPG levels with LGA, however, in the context of Primary Health Care (PHC) where most patients with DM and AH are diagnosed and accompanied, we have not found many studies. Therefore, this study aimed to investigate the association between HbA1c and FPG with the probability of LGA in adult and elderly individuals with DM and AH, within the scope of PHC.

## Methods

### Study design and population

This was a cross-sectional study with adult and elderly individuals previously diagnosed with DM and/or AH registered in 16 (88.9%) PHC services from a municipality in Zona da Mata, Minas Gerais, Brazil, between August 2017 and April 2018.

In 2017, the 16 PHC services in the municipality mentioned, had 6,621 individuals registered and monitored by the PHC, diagnosed with DM and/or AH. Through a sample calculation (Statcalc, Epi-Info, version 7.2), a minimum sample (reference population) of 719 individuals was calculated to identify the prevalence of CKD hidden in this population.

Despite the minimum sample being 719 individuals, we managed to recruit 841 participants, who agreed to participate in the study after clarification. The recruit participants aged 18 years or older and diagnosed with DM and/or AH accompanied by PHC were recruited, who agreed to participate in the study after clarification. We excluded individuals who had severe clinical conditions, those with an established CKD diagnosis, users who abuse alcohol and / or other drugs, individuals unable to move for data collection and pregnant women.

Data from 104 participants who showed albuminuria values above 30 mg / g were disregarded, resulting in a final sample of 737 individuals for the current analysis.

### Data collection and measures

Sociodemographic, lifestyle and clinical data were collected through a semi-structured questionnaire^8^. Weight, in kilograms, was obtained using an electronic scale, with a capacity of 150 kg and a division of 50 g, and height was measured in meters using a portable anthropometer, consisting of a metal platform for positioning individuals and a removable wooden column containing millimeter tape and a reading cursor, according to the techniques of Jelliffe^9^. The Body Mass Index (BMI) was calculated by dividing weight by height square and classified according to World Health Organization (WHO) (2000) criteria for adults and Lipschitz (1994) for the elderly^10,11^.

Waist circumference (WC) and hip perimeter (HP) were measured in centimeters (cm) with an inextensible measuring tape. The WC was obtained immediately above the iliac crest and the HP at the point of greatest hip volume. The values of WC were classified according to the probability for chronic non-communicable diseases according to WHO^10^. The waist-to-hip ratio (WHR) was calculated by the ratio between the values of WC and HP. The WHR values were classified according to cardiovascular probability according to the WHO^10^. Blood pressure was measured by a trained professional with a mercury sphygmomanometer on the left arm, with the subject in a sitting position.

The collections and analyzes of biological materials (blood and urine) were performed by a single accredited laboratory. With prior guidance, participants collected a single sample of the first morning urine for analysis of urinary albumin (mg / dL) and creatinine (mg/dL). UACR was calculated as milligram (mg) of urinary albumin excreted per gram (g) of creatinine and divided into quartiles. The highest quartile (≥ 13 mg/g) was adopted as the threshold for UACR, classifying these individuals as having LGA, without corrections for gender, because there were no differences between genders with regard to stratification of in albuminuria quartiles. The glomerular filtration rate (GFR) (mL/min/1.73 m^2^) was obtained using the formula CKD-EPI (Chronic Kidney Disease Epidemiology Collaboration) and considered to be reduced when < 60 mL/min/1.73 m^2^^1^.

HbA1C was performed using the High-Performance Liquid Chromatography (HPLC) method, certified by the National Glycohemoglobin Standardization Program USA (NGSP). For transporting the material, 2.5 mL of EDTA whole blood was used, cooled to room temperature. The test was performed using the Bio-Rad VARIANT II TURBO HbA1c instrument with the reagent from the same supplier. The ACR was performed using the Turbidimetry method, with 0.5 mL of urine, with transport refrigerated for 7 days or frozen for 30 days. The test was carried out by Abbott's Architect C8000 with Abbott's ready-to-use > 95% liquid reagent.

Blood samples were collected through venipuncture, after 12 h of overnight fasting performed by patients. The following biochemical parameters were analyzed: total cholesterol (TC) (mg/dL) and the fraction of high-density lipoprotein (HDL-c) (mg/dL), triglycerides (TG) (mg/dL), FPG (mg/dL) and HbA1c (%). HbA1c values were classified as < 5.7% (normoglycemia); 5.7 to 6.4% (pre-diabetes or increased probability for diabetes) and ≥ 6.5% (established diabetes), according to the laboratory criteria adopted by the Brazilian Diabetes Society (2019–2020)^12^.

### Statistical analysis

Continuous variables were presented as means and standard deviations or medians and interquartile ranges for asymmetric variables. Categorical variables were expressed in absolute and relative frequencies. The normality of the data was assessed by the Kolmogorov–Smirnov test.

The differences between the three categories of HbA1c were analyzed by one-way analysis of variance (ANOVA) or Kruskal–Wallis for continuous variables and by Pearson's chi-square test for categorical variables. Associations of LGA levels with each increase of 1-SD (standard deviation) in glycemic indicators (FPG and HbA1c) were examined by linear regression at different levels of adjustment, with LGA transformed into a log before analysis.

Logistic regression models were used to assess the association between HbA1c levels and LGA (UACR ≥ 13 mg/g). The probability of LGA in relation to each HbA1c classification was explored in the adjustment for age, sex, alcohol consumption, BMI, WC and serum values of TC, HDL-c, TG and FPG.

The tests were two-tailed and *p* values < 0.05 were considered statistically significant in the multivariable model. The magnitude of the associations was assessed by Odds Ratio (OR) with 95% confidence intervals (CI). Statistical analyzes were performed using the SPSS 20.0 (Statistical Package for the Social Sciences) program.

### Ethical approval

The study is part of a larger project^7^, which followed the ethical precepts of Resolution 466/2012 of the National Health Council, being approved by the Ethics Committee of the Federal University of Viçosa (Opinion: 1203. 173, CAAE: 47356115.3. 0000.5153). The informed consent was obtained from all subjects. All methods were performed in accordance with the relevant guidelines and regulations.

## Results

### Characteristics of the participants

Table 1 shows the demographic and cardiometabolic characteristics of the study population by categories of HbA1c. Accompanying the increase in HbA1c levels, there was a significant increase (*p* < 0.05) in LGA urinary, a progression in the age of participants and in the prevalence of individuals with an isolated diagnosis of DM or with a diagnosis of DM and AH concomitantly.

**Table 1:** General characteristics of participants according to levels of glycated hemoglobin.

	Total	HbA1c levels			
		< 5.7% Normoglycemia	5.7–6.4% Pre-diabetes	≥ 6.5% Diabetes	*p*
**Participants**	737	226 (30.7%)	283 (38.4%)	228 (30.9%)	
**Age (years)**	62.0 [54.0 – 69.0]	60.0 [52.9 – 67.0]	63.0 [54.0 – 70.0]	63.0 [54.5 – 69.0]	< 0.05
**Sex**					0.51
Male	274 (37.2%)	91 (40.3%)	102 (36.0%)	81 (35.5%)	
Female	463 (62.8%)	135 (59.7%)	181 (64.0%)	147 (64.5%)	
**Color / Ethnic**					0.17
Black	160 (23.6%)	43 (20.4%)	59 (23.2%)	58 (27.1%)	
White	225 (33.1%)	78 (37.0%)	74 (29.1%)	73 (34.1%)	
Brown/Yellow/ Indigenous	294 (43.3%)	90 (42.7%)	121 (47.6%)	83 (38.8%)	
**Comorbidities**					< 0.05
Hypertension only	444 (60.2%)	203 (89.8%)	200 (70.7%)	41 (18.0%)	
Diabetes only	52 (7.1%)	5 (2.2%)	8 (2.8%)	39 (17.1%)	
Hypertension and Diabetes	241 (32.7%)	18 (8.0%)	75 (26.5%)	148 (64.9%)	
Alcohol use (yes)	181 (26.7%)	66 (31.1%)	65 (25.8%)	50 (23.5%)	0.19
Current smoker (yes)	76 (11.3%)	30 (14.3%)	29 (11.5%)	17 (8.1%)	0.34
**Biochemical parameters**					
LGA (mg/g)	5.0 [2.0–8.0]	4.0 [2.0–7.0]	5.0 [3.0–8.0]	5.0 [3.0–11.0]	< 0.05
FPG (mg/dL)	97.0 [87.0–122.0]	88.0 [83.0–94.0]	95.0 [87.0–106.0]	141.0 [118.5–184.5]	< 0.05
GFR (mL/min/1.73m^2^)	84.5 ± 19.3	86.9 ± 19.6	82.8 ± 18.7	84.2 ± 19.7	0.056
TC (mg/dL)	191.2 ± 40.0	199.1 ± 40.77	193.5 ± 39.0	180.7 ± 38.3	< 0.05
HDL-c (mg/dL)	49.0 [42.0–59.0]	52 [44.0–61.0]	48 [41.0–60.0]	47.5 [40–57.5]	< 0.05
TG (mg/dL)	126.0 [95.0–171.0]	115.0 [88.0–157.0]	129.0 [98.0–173.0]	130.0 [101.0–179.0]	< 0.05
**Anthropometric parameters**	496 (73.3%)	146 (68.9%)	187 (74.2%)	163 (76.5%)	
SBP (mmHg)	130.0 [120.0–140.0]	130.0 [120.0–140.0]	130.0 [120–140]	130.0 [120–140]	0.584
DBP (mmHg)	80.0 [80.0–90.0]	80.0 [77.0–90.0]	80.0 [78.0–90.0]	80.0 [80.0–90.0]	0.816
BMI (Kg/m^2^)	28.2 [24.8–31.8]	27.1 [23.43–30.7]	28.7 [25.3–32.5]	28.3 [25.3–32.2]	< 0.05
WC (cm)	93.6 ± 11.2	90.5 ± 10.1	94.6 ± 11.8	95.2 ± 11.0	< 0.05
WHR	0.90 [0.85–0.96]	0.88 [0.83–0.93]	0.90 [0.85–0.96]	0.93 [0.86–0.97]	< 0.05

Data were expressed as means ± SD or median (IQR) for continuous variables and numbers (percentages) for categorical variables. The *p* values were calculated using the Annova test and kruskal kal wals for continuous variables and χ2 test for categorical variables.

LGA, low-grade albuminuria; BMI, body mass index; GFR, glomerular filtration rate; FPG, fasting blood glucose; HbA1c, glycated hemoglobin A1c; HDL-c, high density lipoprotein cholesterol; TG, triglycerides; WC, waist circumference; WHR, waist-to-hip ratio.

As HbA1c levels progressed, there was also a progressive reduction in HDL-c and TC levels and a progressive increase in TG and FPG levels. It was also evidenced the occurrence of overweight or obesity through BMI and a gradual increase in cardiovascular probability by the values of WC and WHR, accompanying the increase in HbA1c levels. It was observed that individuals without a diagnosis of DM had HbA1c levels that classified them as pre-diabetes or with DM.

### Association of LGA with Hb1Ac and FPG

Table 2 shows a significant association of LGA in the univariate analysis with FPG and HbA1c in model 1, remaining significant even after adjusting for confounding factors (age, sex, alcohol consumption, TC, HDL-c, TG, BMI and WC) in the multivariate analysis of model 2 (*p* < 0.001). For each increase in a glucose SD (50.7 mg/dl) we have an increase of 6.8 mg/g of LGA. And for each increase in a SD of HbA1c (1.57%) we have an increase of 7.3 mg/g of LGA.

**Table 2:** Association of albumin-to-creatinine ratio for each increase of 1-SD in the levels of HbA1c and FPG.

	Model 1		Model 2		Model 3	
	β ± SE	*P*	β ± SE	*p*	β ± SE	*p*
FPG*	6.70 ± 2.00	0.001	6.80 ± 2.10	0.001	2.30 ± 3.20	0.482
HbA1c**	7.90 ± 1.80	< 0.001	7.30 ± 2.00	< 0.001	5.60 ± 03.10	0.068

Model 1: Not adjusted.

Model 2: Adjusted for age, sex, alcohol consumption, TC, HDL-cholesterol, TG, BMI and WC.

Model 3: * Subsequently adjusted for HbA1c in addition to the model 2 variables; ** Subsequently adjusted for FPG in addition to the model 2 variables.

SD, standard deviation; β, regression coefficient; SE, standard error; BMI, body mass index; FPG, fasting plasma glucose; HbA1c, glycated hemoglobin A1c; HDL-c, high density lipoprotein cholesterol; TG, triglyceride; WC, waist circumference.

However, posterior adjustment for HbA1c and FPG (model 3) reduced to zero the association of LGA with FPG (*p* = 0.170) and with HbA1c (*p* = 0.379), respectively.

### Association of LGA with HbA1c levels

**Figure 1:**
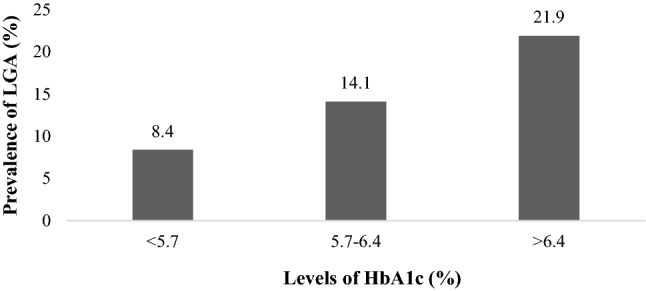
Prevalence of low-grade albuminuria (LGA) according to the levels of glycated hemoglobin (HbA1c).

The occurrence of a significant gradual increase (*p* < 0.001) in the prevalence of LGA following the progression of HbA1c levels (< 5.7%; 5.7–6.4% and > 6.4%) was observed (Fig. 1).

When assessing the relationship between LGA probability and HbA1c levels, taking normoglycemia as the reference category, a significant association was observed in all categories of HbA1c in the univariate analysis (model 1). After the first adjustment for confounding factors (model 2), only participants with HbA1c levels ≥ 6.5% were more likely to also show LGA. When adjusted for FPG (model 3), there was no significance (*p* = 0.359) (Table 3).

**Table 3:** Association of the probability of low-grade albuminuria (LGA) with the levels of HbA1c.

Models	Levels of HbA1c (%)			*p*
	< 5.7 Normoglycemia	5.7–6.4 Pre-diabetes	≥ 6.5 Diabetes	
1	1.00	1.793 (1.007–3.192)	3.06 (1.739–5.384)	< 0.05
2	1.00	1.429 (0.773–2.642)	2.428 (1.322–4.459)	< 0.05
3	1.00	1.372 (0.740–2.544)	1.687 (0.818–3.482)	0.359

ORs (95% CI).

Model 1: Not adjusted.

Model 2: Adjusted for age, sex, alcohol consumption, TC, HDL-c, TG, BMI and WC.

Model 3: Adjusted for FPG in addition to the variables in model 2.

BMI, body mass index; FPG, fasting plasma glucose; HbA1c, glycated hemoglobin A1c; HDL-c, high density lipoprotein cholesterol; TG, triglyceride; WC, waist circumference.

## Discussion

Our study assessing the relationship between the levels of HbA1c and FPG with LGA in a key group of patients diagnosed with DM and/ or HA, accompanied by PHC services. In Brazil, these two clinical conditions together represent 65% of the primary causes of ESKD^6^. The presence of albumin in the urine is a common, but not uniform, finding in CKD, and LGA can be used as a parameter in the initial assessment and monitoring in populations at probability for this disease^1^. The study by Garg et al.^13^ makes this clear when reporting in their findings that, when albuminuria and renal failure were considered together in different segments of the population, participants with GFR less than 30 mL/min/1.73 m^2^ did not demonstrate albuminuria, and 34% of diabetics and 63% of non-diabetic hypertensive patients with GFR less than 30 mL/min/1.73 m^2^ did not demonstrate albuminuria.

Among the possible pathophysiological mechanisms involved in the excretion of albumin, a plasma protein synthesized by the liver, are inflammation, endothelial dysfunction and capillary permeability^14^. The increase in albuminuria may be associated with elevated systolic blood pressure, hypertriglyceridemia, HDL-c reduction, obesity, metabolic syndrome and insulin resistance^1,14,15^. However, urinary albumin levels are fluctuating and LGA must be interpreted in the context of the potential underlying etiology and as a dynamic vascular system, which reflect biological complexity albumin excretion. LGA associated with inflammation may reflect a pathological process distinct from LGA associated with insulin resistance or AH^13^.

In another recent study that followed 37,091 individuals for 10 years, LGA was an independent risk factor for incident AH and mortality from cardiovascular disease, but not for the incidence of DM, during the same follow-up period^2^. However, in diabetic adults, LGA has been associated with peripheral arterial disease and poor memory performance in elderly diabetics without dementia^16,17^, in addition to being an early marker for the detection of arterial stiffness^18^.

The significant association between HbA1c levels and the prevalence of LGA has also been demonstrated in a previous study with Chinese middle-aged adults^3^. The main difference from the current study was that they found an independent correlation between HbA1c with LGA, but no similar association for FPG or blood glucose after two hours of glucose overload. It is worth mentioning the divergence between the sample populations of the studies, as well as the observation of a possible collinearity between the variables HbA1c and FPG in the present study, since, despite being different biochemical tests, both measure the presence of glucose in the blood. Another study finds that the prevalence of prediabetes according to FPG/2 h-PG among individuals with normal HbA1c is considerably high, and the prevalence of moderately increased albuminuria in this group is significantly elevated^19^.

A question that arises is whether glycemic control would provide better primary and secondary outcomes related to long-term albumin excretion. In this sense, Qingrong Pan et al. (2018) demonstrated that LGA (UACR 10-30 mg/g) was associated with metabolic factors before treatment and that the use of metformin or acarbose significantly reduced albuminuria in this group^20^. A systematic Cochrane review also suggested that people who receiving intensive glycemic control (HbA1c < 7%) for treatment of DM compared to less rigorous control (HbA1c > 7%) experienced small clinical benefits in relation to the onset and progression of microalbuminuria and non-fatal acute myocardial infarction^21^.

Some limitations of this study must be mentioned. First, due to the cross-sectional characteristic of this study, we have not drawn any conclusions about cause and effect between HbA1c and FPG in relation to LGA. Secondly, the fact that we did not measure 24-h albuminuria, considered the gold standard^1^, however, the use of urine in a simple and unique test has good agreement and can be a reliable alternative^2,3^. Third, we did not calculate specific LGA thresholds for the sexes, with the possibility of subtle differences between them. In addition, blood glucose after two hours of glucose overload and serum insulin for calculating the HOMA index, best markers for assessing insulin resistance, were not analyzed^1^.

## Conclusions

In conclusion, the study demonstrated that HbA1c and FPG were significantly associated in a collinear manner with an increased risk of LGA in adults and elderly individuals with DM and AH, accompanied by PHC. The recognition of this association can contribute to the preventive management of patients in the risk group for the DRC, encouraging changes in lifestyle and the control of modifiable factors.

However, in order to better evaluate the therapeutic properties, further long-term prospective studies are needed to compare the levels of HbA1c and FPG, relating them to the development of LGA and to important primary outcomes, such as progression to ESKD and mortality.
